# Autoimmune antibodies in first-episode psychosis with red flags: A hospital-based case-control study protocol

**DOI:** 10.3389/fpsyt.2022.976159

**Published:** 2022-10-05

**Authors:** Jianjun Wang, Xuan Liu, Jie Lian, Haotao Zheng, Dongbin Cai, Haobin Cai, Dan Zhou, Songjun Lin, Fanxin Kong, Xiude Qin, Jianqiang Bi

**Affiliations:** ^1^Department of Neurology and Psychology, Shenzhen Traditional Chinese Medicine Hospital, Shenzhen, China; ^2^The Fourth Clinical Medical College, Guangzhou University of Chinese Medicine, Shenzhen, China; ^3^Global Clinical Scholars Research Training, Harvard Medical School, Boston, MA, United States; ^4^Department of Laboratory Medicine, Shenzhen Traditional Chinese Medicine Hospital, Shenzhen, China; ^5^Department of Public Health, Shenzhen Kangning Hospital, Shenzhen Mental Health Center, Shenzhen, China

**Keywords:** first-episode psychosis (FEP), autoimmune, autoantibody, red flag, N-methyl-D-aspartate receptors (NMDAR)

## Abstract

**Background:**

Research is increasingly identifying an overlap between psychosis and immunological dysregulation. Certain autoantibodies are being identified in a small but probably relevant subgroup of patients with psychosis. The term “autoimmune psychosis” (AIP) and its corresponding red-flag signs present the opportunity for a new field in psychiatry to promote diagnostic workup and immunomodulating therapy in individual cases.

**Objectives:**

The present protocol aims to determine the seroprevalence of autoantibodies in first-episode psychosis (FEPs) using AIP red flag signs, and to explore the frequency of autoantibody subtypes and potential mediating confounders.

**Methods/design:**

This is a hospital-based case-control study. All participants will be consecutively selected from the main tertiary psychiatric hospital in Shenzhen City, China. Individuals admitted to the psychiatric ward and diagnosed with FEPs will be enrolled. Based on recent consensus, participants with red flags of AIPs will be defined as cases, while the remainder will be matched as controls. Seropositive antibodies will be detected and verified in cerebrospinal fluid (CSF) samples based on the fixed cell-based assay (CBA) method. The propensity score-adjusted odds ratios will be determined to investigate the key mediating confounders regarding autoantibody subtypes and red flag subsets.

**Discussion:**

The results of this study will facilitate the early identification of AIPs in FEP patients using the red flag sign and help identify key mediators that improve the accuracy of diagnostic algorithms. It will have clinical significance to focus on serum antibodies that have been verified in CSF samples, due to its consistency with clinical practices in current psychiatry.

## Introduction

First-episode psychosis (FEP) is a severe condition characterized by delusions, hallucinations, and various negative symptoms, typically occurring for the first time during adolescence (1). As the most critical factor affecting subsequent social functioning, FEPs should be thoroughly investigated at the initial phase. The first and foremost step is to precisely identify the etiology, which is a prerequisite for proper treatment and has evolved vastly over time (2). The dichotomization of organic and functional psychosis has been prevalent for more than a century (3). A recent realization is more useful to categorize it as primary or secondary psychosis (4). This shift is more practical to encourage the discovery of psychiatric symptoms in general medical conditions, where suspecting an underlying medical illness is a logical initial step when encountering psychosis in general medical settings. Recently, the heated debates of revisiting the concepts of pseudo-schizophrenia, secondary schizophrenia, and schizophrenia-like psychosis emphasize the need to be vigilant regarding cases of *de facto* organic psychosis misdiagnosed as schizophrenia and advocate it as the ultimate diagnosis of exclusion in psychiatry (5–7). These historical concepts have never been outdated, and with the development of basic medical science, a new cause-effect relationship is constantly being discovered, such as autoimmune-mediated psychosis (8).

Over the past decades, accumulating human data suggested that immunological abnormalities could contribute to the neurobiology of primary psychotic disorders (9). Since its discovery in 2007, patients with anti-N-methyl-d-aspartate receptor (NMDAR) encephalitis have been observed to manifest prominent psychotic symptoms and to respond well to immunotherapies. As the most discussed antibody relevant to psychotic disorders, 4% of patients with anti-NMDAR encephalitis developed isolated psychiatric symptoms without obvious neurological manifestations before the onset of encephalitis or as a relapse (10). These psychotic manifestations are similar to those seen in primary functional psychiatric disorders (11). Another study revealed that 12 of the 15 patients (80%) with NMDA-R encephalitis presented with prominent psychiatric symptoms (12), and that 34–53% of patients with autoimmune encephalitis (AE) were initially referred to a psychiatric department (12, 13). However, the prevalence varied considerably in different studies (14). A meta-analysis found that the NMDAR antibody could be detected in 1.5–8% of psychotic patients with sufficient variations in methodological and patient factors (14, 15). Meanwhile, a large-scale study failed to detect the NMDAR antibody in patients with schizophrenia (16). Therefore, given the inconsistencies in previous studies (15), it is necessary to develop a more precise diagnostic workup initiated in a small but potentially ideal subgroup of psychiatric patients.

An international consensus has recently proposed a novel list of clinical warning signs, or red flags, allowing clinicians to specifically investigate the early phases of autoimmune psychosis (AIP) in psychiatric practice (8). Examples of clinical red flags suggestive of autoimmune causes include (a) atypical psychotics with additional neurological presentations, (b) tumor, (c) evidence of cognitive decline, (d) decreased level of consciousness, and (e) magnetic resonance imaging (MRI)/cerebrospinal fluid (CSF)/electroencephalogram (EEG) abnormalities (8). However, one small-scale observational study found no NMDAR antibody in patients with FEPs and raised concerns about the clinical utility of warning signs in diagnosing AIP (17). Thus, an optimized diagnostic algorithm of autoimmune FEPs is urgent to address some of the challenges inherent to the early diagnosis of FEPs presenting with these warning signs (9). We propose not to overlook appropriate neurological workups such as MRI, EEG, and CSF biomarkers, but rather to apply the consensus criteria more broadly to formulate hierarchical differential diagnostic considerations underlying autoimmune involvement among organic etiologies. The optimized diagnostic algorithm could facilitate better differentiations from AE and thus avoid misdiagnosis. Further, full consideration of autoimmune characteristics could promote immunosuppressive treatment for conventional, atypical, and refractory psychoses (8, 18), thereby introducing a new treatable choice for these small but potentially curable patients.

## Objectives

### Main objective

The main objective is to determine if the prevalence of any autoantibodies in the case group is significantly higher than that of the control group.

### Secondary objectives

The secondary objectives are (1) to determine whether serum and CSF autoantibody positivity is different and (2) to explore antibody subtypes and red flag subsets that cause differences.

## Hypotheses

Compared with FEPs without red flag signs, FEPs with red flags have significantly higher rates of autoantibody positivity. Serum antibodies are consistent with CSF antibodies in some antibody subtypes and red flag subsets.

## Research design and methodology

### Participants and procedure for recruitment

The study will enroll individuals with FEP admitted to the inpatient psychiatric department of Shenzhen Kangning Hospital, which is the main tertiary referral hospital for psychiatric disease in Shenzhen city with a population of 20 million. Face-to-face interviews will be administered by a clinical researcher to illustrate the research procedure. Written informed consent will be obtained from the patients as well as their legal guardians.

### Study design

**FIGURE 1:**
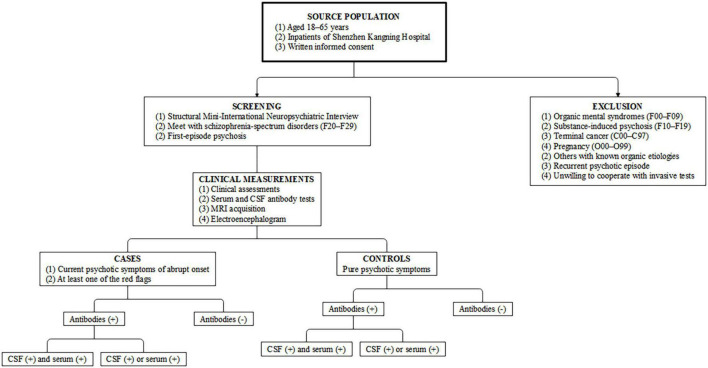
Study design and case–control definitions. CSF, cerebrospinal fluid; MRI, magnetic resonance imaging.

An individually matched hospital-based case–control design will be used in this study. All participants will be selected from individuals admitted to the psychiatric ward for medical management, but only patients with FEP will be included for further evaluation. According to the recent consensus on red flags of suspected AIP (8), participants with at least one sign will be categorized into the case group, while all other patients will be enrolled as controls. An overview of the study design and case definitions are presented in Figure 1.

### Inclusion and exclusion criteria

Participants aged 18–65 years and that meet the International Classification of Diseases 10th Revision (ICD-10) criteria for schizophrenia-spectrum disorders (F20–F29) will be eligible for inclusion in this study (19), but only patients with FEP will be included for further evaluation. A structured Mini-International Neuropsychiatric Interview (MINI) interview (20) will be conducted by two trained researchers to determine the diagnosis. Specifically, the FEP cases should present with current psychotic symptoms of abrupt onset (rapid progression of < 3 months) and with at least one of the red flags listed in Table 1. The rest of the participants from the same cohort will be considered as controls if they present with pure psychiatric symptoms without any red flags.

**TABLE 1:** Red flags for psychosis of suspected autoimmune origin.

Item	Red flags
Clinical characteristics	(1) Tumor; (2) Catatonia or dyskinesia; (3) Adverse response to antipsychotics with rigidity, hyperthermia, or raised creatine kinase; (4) Severe or disproportionate cognitive dysfunction; (5) Decreased level of consciousness; (6) Seizures; (7) Abnormal blood pressure, temperature, or heart rate.
Test results	(8) CSF pleocytosis of > 5 white blood cells per μL, or CSF oligoclonal bands or increased IgG index; (9) MRI abnormalities on bilateral medial temporal lobes; (10) Electroencephalogram encephalopathic changes.

CSF, cerebrospinal fluid; IgG, Immunoglobulins G; MRI, magnetic resonance imaging.

Patients who are physically unable or too unwell to participate in the study and those already diagnosed with organic mental syndromes (F00–F09) will be excluded from the study. Further, patients with substance-induced psychosis (F10–F19), terminal cancer (C00–C97), or pregnancy (O00–O99) will be also excluded. As the focus of this study is on FEPs, cases and controls with a recurrent psychotic episode will be excluded from the study.

### Sample size and statistical approach

The sample size was calculated using the Power Analysis and Sample Size software (PASS) version 11.0 (NCSS Statistical Software, Kaysville, Utah, USA). Based on a seroprevalence of 3% in the control group (21), and utilizing a Chi-squared test for a correlated proportion of 1:5, a sample size of 181 cases and 905 controls will have 80% power to detect an odds ratio of 3.0 at a *p* = 0.05 significance level.

### Recruitment

The study is expected to consecutively recruit participants over a 2-year period from November 2022 to October 2024. Interviews will be conducted by trained investigators in the participant’s preferred language (Cantonese or Mandarin). Interviewers will not be blinded to the case/control status of the participant. To minimize interviewer bias, investigators will be asked to follow the standard MINI interview. The supervisor (JW) will regularly shadow interviewers to ensure that the interviewers adhere to the procedure.

The authors assert that all procedures contributing to this work comply with the ethical standards of the relevant national and institutional committees on human experimentation and with the Helsinki Declaration of 1975, as revised in 2008. All procedures involving human patients have been approved by the Ethics Committee of Shenzhen Kangning Hospital (K2022-016-01). Written informed consent to participate in this study will be obtained from the participants and their legal guardians.

### Measurements

#### Routine assessments

ICD-10 diagnosis will be re-evaluated by two trained investigators using the structured MINI interview. The degree of psychotic symptoms will be measured using the Positive and Negative Syndrome Scale (PANSS) (22). The overall functional disability will be assessed *via* the Global Assessment of Functioning (GAF) (23). Data on patient sex, age at onset, duration of disease, symptoms, neurological examination, and treatment will be obtained from the chart record.

Common organic disease associated with psychiatric symptoms will be evaluated. The level of testosterone, estrogen, progesterone, prolactin, and thyroid hormone will be measured to exclude endocrine diseases. Urinalysis and infectious serologies will be used to exclude drug abuse, syphilis, Lyme disease, acquired immunodeficiency syndrome, and viral hepatitis. A wide spectrum of vitamins such as vitamins B1, B3, B6, and B12 will be used to exclude beriberi, pellagra, and Wernicke-Korsakoff syndrome. Generic antibodies will be used to exclude classic autoimmune diseases such as systemic lupus erythematosus, Sjogren’s syndrome, and Behcet’s syndrome.

#### Plasma and cerebrospinal fluid samples

Plasma will be collected for all participants, while CSF samples will be only collected in case group. The standard venipuncture in the cubital region will be followed using a butterfly needle and vacutainer needle holder. The CSF puncture will be performed with a spinal needle between the 4th and 5th lumbar vertebrae in lateral position. The tube will be labeled with the anonymous subject barcode and kept in a refrigerator at 4 degrees centigrade. Then, the plasma samples will be centrifuged at 3,000 g for 8 min within 2 h. All samples will be aliquoted in 0.5 mL Eppendorf safe-lock tubes and stored at minus eighty degree centigrade until tested.

#### Antibody testing

Serum and CSF autoantibodies will be detected using a commercially available biochip manufactured by Guangzhou KingMed Diagnostics Group (Guangzhou, China). The fixed cell-based assay (CBA) consists of HEK 293 cells transfected with plasmids encoding both intracellular and extracellular antibody antigens (Table 2). The analytical technician will be blinded to the case status. Two independent masked assessors will classify each sample as positive or negative based on the surface immunofluorescence intensity, which is directly compared with that of non-transfected cells and control samples. Once verified, the positive samples will then be serially diluted from 1:10 to 1:1,000 to determine the titer. The final titer is defined as the dilution value of the sample whose specific fluorescence is almost clearly identifiable and expressed as the corresponding dilution value. This preparation follows an established method and has been described in detail previously (24).

**TABLE 2:** Antibodies tested in serum and CSF samples.

Samples	Antibodies
Serum	NMDAR, GAD65, LGI1, CASPR2, AMPA1, AMPA2, GABAB, IgLON5, DPPX, GlyR, MGluR5, MGluR1, Neurexin-3a
CSF	NMDAR, GAD65, LGI1, CASPR2, AMPA1, AMPA2, GABAB, IgLON5, DPPX, GlyR, MGluR5, MGluR1, Neurexin-3a

NMDAR, anti–N-methyl-D aspartate receptor consisting of NR1 and NR2B subunits; GAD65, glutamic acid decarboxylase 65; LGI1, leucine-rich glioma inactivated protein 1; CASPR2, contactin-associated protein-like 2; AMPAR1, α-amino-3-hydroxy-5-methyl-4-isoxazolepropionic acid receptor subunit 1; GABABR, γ-aminobutyric acid-B receptors; IgLON5, immunoglobulin-like cell adhesion molecule 5; DPPX, dipeptidyl-peptidase–like protein 6; GlyR, glycine receptor; MGluR5, metabotropic glutamate receptor subunit 5; CSF, cerebrospinal fluid.

#### Routine cerebrospinal fluid tests

The routine CSF parameters will be analyzed with reference to Reibergrams procedures (25, 26), including cell count, total protein, albumin, and CSF-specific oligoclonal bands (OCB). Immunoglobulins A, G, and M (IgG, IgM, IgA) and albumin quotient (QAlb) will be measured by immunonephelometry.

#### Magnetic resonance imaging acquisition

MRI scanning will be conducted for all participants on a 3T scanner (GE medical system, MR750) with a 32-channel head-coil. The scanning parameters are as follows: (1) T1 images: repetition time = 8.656 ms, echo time = 3.22 ms, inversion time = 450 ms, flip angle = 12°, matrix size = 256 × 256, slice thickness = 1 mm, voxel size = 1 × 1 × 1 mm^3^, and sections = 152. T2 FLAIR: repetition time/echo time ratio = 9,000/92.544 ms, flip angle = 160°, matrix size = 256 × 224, slice thickness = 6.5 mm, and voxel size = 0.47 × 0.47 × 6.5 mm^3^. Diffusion weighted images: 25 diffusion images with b = 1,000 s/mm^2^ and 4 non-diffusion-weighted images (b = 0 s/mm^2^), repetition time = 8,000 ms, echo time = 101.1 ms, field of view = 256 × 256 mm^2^, matrix size = 256 × 256, slice thickness = 4 mm, and slice number = 38.

### Statistical analyses

All analyses of primary and secondary variables will be carried out using the intention-to-treat principle. Continuous data following a normal distribution will be presented as mean and standard deviation, and independent sample *t*-test or one-way analysis of variance will be used to determine significant differences among groups. Continuous data with a non-normal distribution and ordinal variables will be presented as median with quartiles and compared using the Mann–Whitney U or Kruskal–Wallis test. Absolute numbers and percentages will be used for categorical variables, and the Chi-square test will be used to compare the group differences.

Descriptive statistics will be used to characterize demographic, behavioral, and clinical variables for the study sample. Multivariate modeling to address our aims will be based on methods previously described (27). The seroprevalence of any autoantibodies in FEPs, defined as the positive dilution value based on the CBA method, will be calculated using matched ORs from a conditional logistic regression model with the autoantibody positivity as exposure and case/control status as the outcome. Then, a propensity score algorithm will be used to match cases to controls at a 1:1 ratio. The propensity score-adjusted odds ratios for the exposure of interest will then be determined. All demographic variables, laboratory values, and clinical characteristics will be screened for inclusion in each model. The propensity score will be included as a covariate. Variables with a significant association with the exposure of interest of *p* < 0.20 and a number of events > 10 will be included in the model (28). In addition, we will conduct a *post hoc* power analysis, setting the significance at a type I error of 5% (two-sided). Statistical analyses will be performed using Statistical Package for the Social Sciences (SPSS) statistics for Windows version 21.0 (International Business Machines Corporation, Armonk, New York, United States).

## Discussion

There is an urgent clinical and societal need to improve outcomes of FEPs. Excluding organic causes in every patient presenting with psychosis syndrome is one of the primary and preferred strategy. Recent advances in autoimmune knowledge have opened more opportunities for ameliorating outcomes of FEPs during its early stages. With the increasing identification of immunological abnormalities in patients with primary psychoses (2, 3), this study combines autoimmune antibodies in serum and CSF samples and the red flags of the consensus to formulate an optimized screening algorithm for AIPs. However, before taking advantage of these modern developments, we should always bear in mind the primary rules in this area. First, psychiatric symptoms are common among individuals with neurological disease, e.g., catatonia in patients with anti-NMDAR encephalitis (29), and psychiatric symptoms in paraneoplastic autoimmune limbic encephalitis (30). Second, whenever antibodies are measured as biomarkers for AIPs, the results should be interpreted with caution, as there will always be false positives and false negatives. Moreover, it is essential to provide CSF examination in suggestive cases because serum alone is not sufficiently diagnostic (31). Finally, it is becoming clear that there exist even more unknown antibodies with pathogenic relevance to the spectrum of psychiatric syndromes potentially responding to immunotherapies (32).

An increasing number of studies point to the overlap between psychosis and pathological processes associated with immunological dysregulation. Notably, the recent discovery of antibodies against synaptic and neuronal cell membrane proteins such as anti-NMDAR provides more direct evidence of the etiological connection between autoimmunity and the subsequent hazard of psychosis (9). Kelleher et al. found a 3.5% NMDAR seroprevalence in a cohort of patients with FEP, and only one case reached a definite diagnosis of NMDAR encephalitis (21), which supported the necessity of the recently suggested term “autoimmune psychosis.” Currently, an increasing number of subtypes of autoantibodies have been found to be involved in prominent psychiatric features (33), including antibodies against intracellular antigens (e.g., Hu, Ma2, GAD), antibodies against synaptic receptors (e.g., NMDA receptor, AMPA receptor, dopamine 2 receptor, mGluR5, GABAA/B receptor), and antibodies against ion channels and other cell-surface proteins (e.g., LGI1, CASPR2, DPPX, MOG, Aquaporin 4, GQ1b). Based on the newly proposed international consensus on the diagnosis of AIP (8), we propose to determine the seropositive prevalence of autoantibodies related to the so-called red flag symptoms. We hypothesize that FEP patients with red flags have a much higher seroprevalence of autoantibodies than the controls.

A generalized antibody analysis could follow a series of standardized steps. First, it would be the most prevalent antibodies against cell surface antigens (e.g., NMDAR) and intracellular antigens (e.g., GAD65). Less frequent antineuronal antibodies (e.g., DPPX) can be investigated in the second step, when the initial screening is negative and/or some suggestive clinical factors prevail. In addition, it is also important to bear in mind the existence of seronegative but probable AIP in clinical practice, as it has been reported that in a subset of drug-resistant primary psychosis, some patients respond well to immunotherapies (9, 32). Meanwhile, the psychiatric characteristics of these pathogenic autoantibodies are highly variable (10), making it necessary to screen for a wide spectrum of autoantibodies and to explore the relevance of each subtype. Tissue-based assays on brain slices of rodents may be an advanced method for previously unknown antineuronal antibodies in such cases. Based on the hierarchical frequency of relevant autoantibodies, an optimized screening algorithm for FEPs could be formulated (34).

There are many methodological issues worthy of attention (15). The most influential factor attributed to the clinical inconsistencies is the assay detection method. Generally, immunoglobulins are classified into three subclasses: IgA, IgG, and IgM isotypes, which can be detected by either Enzyme-Linked Immunosorbent Assay (ELISA) or CBA approaches. The latter can be further divided into fixed CBA or live method. For example, when compared to studies using a fixed CBA, studies using the live approach have a much higher prevalence rate in psychosis (15). In the present study, we propose the live CBA method to ensure the higher accuracy. In addition, potential heterogeneities within the sample due to varied clinical characteristics could also affect the outcome. To address this, we have selected a representative sample with a validated diagnostic assessment and restricted the sample to only FEP patients with detailed descriptions of illness severity. Furthermore, we implemented a strictly defined propensity score match to control for confounding variables. We hypothesize that FEPs with red flags would show significantly increased seropositivity for specific antibodies after adjusting for individual differences.

Further limitations should also be noted. First, we only collect CSF samples in the case group, which may miss the CSF antibody-positive cases in the control group. However, given the ethical requirements in current psychiatric practice, further study is needed to address this issue in a more practical way. Another concern is the recruitment. Given that psychosis with suspected autoimmune origins is a rare condition, a sufficiently large sample size is required to ensure adequate statistical power. Therefore, the project will be conducted in 2 years to ensure sufficient samples. Finally, a case–control study is a preliminary design for studying rare diseases, and it is important to consider the potential issues regarding selection bias.

The outcome of this study will assist to facilitate early identification of AIPs using red flag signs and to develop an optimized screening algorithm for patients with FEP. It could potentially expand into a new field of immunotherapeutics for a small but crucial subgroup of autoantibody-mediated psychosis. We will publish our findings in international journals and will also disseminate the work at national and international conferences and in local policy fora.

## Ethics statement

All procedures involving human patients were approved by the Ethics Committee of Shenzhen Kangning Hospital (K2022-016-01). The patients/participants provided their written informed consent to participate in this study.

## Author contributions

JW, XL, HZ, DZ, HC, FK, XQ, and JB conceived of the protocol, performed the literature search, and conceived of the initial protocol. JW, XL, and DC wrote the manuscript. JW, XL, FK, XQ, and JB contributed to the design of the final protocol. HZ, DC, HC, DZ, and SL were responsible for piloting the survey. FK, XQ, and JB were responsible for supervising the data collection. All authors provided critical evaluation and revision of the manuscript and have given final approval of the manuscript accepting responsibility for all aspects.
